# Biofuels and the role of space in sustainable innovation journeys^☆^

**DOI:** 10.1016/j.jclepro.2013.07.057

**Published:** 2014-02-15

**Authors:** Sujatha Raman, Alison Mohr

**Affiliations:** Institute for Science & Society (ISS), School of Sociology & Social Policy, University of Nottingham, Nottingham NG7 2RD, United Kingdom

**Keywords:** Biofuel, Agrofuel, Sustainable innovation journey, Spatial order, Sustainability assessment

## Abstract

This paper aims to identify the lessons that should be learnt from how biofuels have been envisioned from the aftermath of the oil shocks of the 1970s to the present, and how these visions compare with biofuel production networks emerging in the 2000s. Working at the interface of sustainable innovation journey research and geographical theories on the spatial unevenness of sustainability transition projects, we show how the biofuels controversy is linked to characteristics of globalised industrial agricultural systems. The legitimacy problems of biofuels cannot be addressed by sustainability indicators or new technologies alone since they arise from the spatial ordering of biofuel production. In the 1970–80s, promoters of bioenergy anticipated current concerns about food security implications but envisioned bioenergy production to be territorially embedded at national or local scales where these issues would be managed. Where the territorial and scalar vision was breached, it was to imagine poorer countries exporting higher-value biofuel to the North rather than the raw material as in the controversial global biomass commodity chains of today. However, controversy now extends to the global impacts of national biofuel systems on food security and greenhouse gas emissions, and to their local impacts becoming more widely known. South/South and North/North trade conflicts are also emerging as are questions over biodegradable wastes and agricultural residues as global commodities. As assumptions of a food-versus-fuel conflict have come to be challenged, legitimacy questions over global agri-business and trade are spotlighted even further. In this context, visions of biofuel development that address these broader issues might be promising. These include large-scale biomass-for-fuel models in Europe that would transform global trade rules to allow small farmers in the global South to compete, and small-scale biofuel systems developed to address local energy needs in the South.

## 1 Introduction

Since the late 2000s, biofuels have been characterised by environmental and development groups as *‘*a big green con’ and a ‘crime against humanity’ (BBC, 2007; Friends of the Earth, 2007). Investigating these concerns, a number of policy agencies have called for biofuels to be sustainable (e.g., OECD/FAO, 2007; Renewable Fuels Agency, 2008). The European Union amended its 2003 Biofuels Directive to include a sustainability clause in the 2009 Renewable Energy Directive, and various certification schemes such as from the Roundtable on Sustainable Biofuels have emerged as mechanisms for demonstrating compliance. ‘Second-generation’ biofuel technologies that would use non-edible feedstocks rather than food crops are also advocated as the future of biofuels (Royal Society, 2008; Suurs and Hekkert, 2009).

In this landscape, it is largely assumed that the problems of biofuels can be addressed by indicators showing how specific sustainability criteria such as food security, human rights, and GHG savings are met in the sourcing of biomass or, in the future, by new technologies. But are these lessons adequate? Could they be addressing the ‘wrong problem’?^1^ Social science work on the recent biofuels controversy (e.g., Journal of Peasant Studies, 2010 special issue on the Politics of Biofuels; Levidow and Paul, 2008; Mol, 2007; Palmer, 2012; Upham et al., 2011; van der Horst and Vermeylen, 2011) suggests biofuels face broader legitimacy problems that cannot be adequately addressed by sustainability indicators alone. Departing from this view, Pilgrim and Harvey (2010) argue that NGOs have campaigned simply for strategic influence and overstated the problems of biofuels, but they do not themselves attempt to assess the sustainability issues. Against this backdrop, we aim to re-assess the history of biofuels in order to distil the key lessons that should be learnt. Taking a longer-term perspective dating back to the late 1970s, the paper complements recent social science work which focuses on the current controversy.

We pose three seldom-raised questions. First, how should we make sense of the ‘riches-to-rags’ journey of biofuels (Sengers et al., 2010)? As recently as the early-mid 2000s, environmental NGOs such as Greenpeace and Friends of the Earth included biofuels in their campaigns for carbon-reducing technologies while renewable policy analysts characterised liquid biofuels as ‘a relatively easy and important carbon-neutral additive to petrol for transport’ (Mitchell and Connor, 2004: 1942), a judgement that is now unlikely to be made without qualification. Second, how much were the challenges of biomass-based fuel anticipated earlier as scientists and energy researchers began to rediscover and promote bioenergy after the oil shocks of the 1970s? This early history of biofuels can shed new light on contemporary dilemmas if we can recover lessons to be learnt or promising options for biofuel development that have been forgotten. Third, how are those advocating biofuels responding and are there any new insights since the controversy first emerged? Overall, UK biofuel policy has not fundamentally changed despite controversy (Palmer, 2010), so there is a need to ensure that the right lessons are drawn from the experience so far.

Mol (2007) provides a clue to understanding the riches-to-rags journey of biofuels. He suggests that debates on the merits of biofuels remained localised so long as production systems were themselves local or national in scope; however, this has changed with the rise of a globally integrated biofuel network. Likewise, van der Horst and Vermeylen (2011) highlight problems arising from global North/South biofuel networks in which crops and oils are produced in poorer Southern regions for biofuel use in the richer North. The spatial structure of biofuel production is therefore implicated as an underlying factor in the current problems of biofuels. In this paper, we aim to investigate this hypothesis more systematically by examining the historical promotion of bioenergy as well as the recent debate.

Our central question is: how has the spatial structure of the technology *and* the reach of its impacts been imagined and understood over time as biofuels have been promoted, challenged and lately, reconstructed in new ways? The paper contributes to recent interest in this journal on the systemic aspects of biofuels as for example, a recent set of articles examining the linkage between biofuels and international trade (Journal of Cleaner Production, November 2009 special issue).

In the next section, we outline our methodology and theoretical framework. Section 3 contains the main findings from our overview of how biofuels have been promoted and assessed since the oil shocks. In Section 4, we conclude by returning to the hypothesis and outlining the lessons for the future of biofuels.

## 2 Framework and methodology: space and the sustainable innovation journey

Research on sustainable innovation journeys or transitions (Geels et al., 2008) provides a starting point for our investigation, though we also draw on related work to flesh out the key role of expectations (Berkhout, 2006) and space (Coenen et al., 2012) in sustainable innovation. The sustainable transitions research tradition shares a focus on the discontinuous, negotiated and uncertain nature of radical technological change in which the interaction between multiple actors, material infrastructures, institutions, social norms, and practices shapes the fate of novel technologies (Coenen and Lopez, 2010). The notion of a journey highlights the importance of agency since socio-technical transitions evolve in ways that cannot be known in advance of the navigation of unexpected turns and struggles.

Within the specific innovation journey approach of strategic niche management (SNM), the *articulation of expectations and visions* to provide direction and legitimate protection of the niche; *building of social networks* to create a community of actors around the technology and facilitate interaction between them; and *learning at multiple levels* are all important for developing new sustainable technologies (Schot and Geels, 2008). While the significance of network-building is fairly self-explanatory, it is worth looking at the concepts of expectations and learning more closely, not least because these are not always defined clearly.

Expectations (often used interchangeably with ‘visions’), or more specifically, technological expectations, have been defined in the sociology of expectations literature which underpins SNM theory as “representations of future technological situations and capabilities” (Borup et al., 2006: 286). They guide and structure innovation activities, attract interest and investment, and (when successful) help create legitimation (Borup et al., 2006). By giving meaning to a specific future which is taken as desirable, specific expectations may also effectively foreclose other possible futures if enacted (Brown and Michael, 2003). Since expectations or visions could take a huge variety of forms including tacit or privately-held views, Berkhout (2006) argues for a clear definition: “*collectively held and communicable schemata that represent future objectives and express the means by which these objectives will be realised*” (p.302). Not all expressions or assumptions of future possibilities or options become ‘expectations’ or ‘future visions’. Expectations must also be publicly communicated, though this might take place by different actors (scientists, engineers, professionals, policymakers, business) in a variety of venues (scientific journal articles, press releases, government or industry documents or other multimedia sites). In addition to the expression of future *objectives*, Berkhout (2006) suggests that expectations presuppose a set of social and institutional relationships (‘*orders’)* and the technological means (‘*technologies’*) for achieving these objectives.

Learning is clearly important for recognising negative impacts of new technologies and responding appropriately. But, what kinds of lessons are drawn? Do we know if they are the right ones? In this context, it is important to consider the argument from human geographers that sustainable transitions are not neutral as they have uneven spatial impacts (Swyngedouw, 2007; Whitehead, 2007) and a politics in which some visions triumph over others (Shove and Walker, 2007). “Particular trajectories of socio-environmental change undermine the stability or coherence of some social groups, places or ecologies, while their sustainability elsewhere might be enhanced” (Swyngedouw, 2007: 37). Hornborg (2008) highlights a process of ‘environmental load displacement’ where civilisations that ‘succeed’ do so “by exporting their environmental problems, and jeopardising ‘sustainability’ elsewhere” (Hornborg, 2008: 103–104). These arguments suggest that there is a strong potential for *spatial unevenness* in the journey of sustainable innovation. Through the articulation of expectations, creation of networks and learning from a process of twists and turns, novel technologies may become successfully embedded in one spatial domain but pose threats at another.

By comparison with time, the role of space is largely neglected in the sustainable transitions literature (but see van Eijck and Romijn, 2008). A recent paper (Coenen et al., 2012) aims to fill this gap by bringing spatial insights of economic geography to bear on sustainability transition theories. The authors critique the focus on national transitions, pointing out that in a globalised context, national visions often rely on resources from wider networks and markets. At the same time, they note the global is not just an external force that exists ‘above’ national or local scales. The construction of scale in environmental projects is itself political (Bulkeley, 2005); different scales overlap and local actors can argue back, so, a ‘local node, global network’ perspective is more appropriate (Coenen et al., 2012). In addition, the notion of institutional orders which Berkhout (2006) argues are part of any configuration of expectations should be recognised as having a *spatial* dimension.

As a technology promoted for sustainable transitions, the case of biofuels is particularly apt for extending this line of analysis on the role of *spatial orders* in sustainability. The paper adopts the innovation journey methodology of examining technology trajectories over a longer time-frame in order to make sense of what has (or has not) been learnt. In particular, we look at how the spatial dimension of biofuels is envisioned, how these visions changed over time and how they compare with the spatial orders of biofuel systems that developed. We use the qualitative social research method of documents as a source of data and analysis (Bryman, 2012). Since the study of expectations around emerging technologies commonly involves looking at their articulation in scientific as well as ‘grey’ literature, we survey articles in the field of energy research since the late 1970s, supplemented by other key academic articles and reports produced by policy, professional and non-governmental organisations (NGOs). Treating these documents as a historical record of how biofuels have been understood and imagined over time, we aim to distil the main themes including ones that have been subsequently ignored. We focus mainly on the UK debate, but as this debate intersects strongly with developments elsewhere, we draw on these where appropriate. In this respect, the paper complements work employing the strategic niche management perspective to examine biofuel experiments in the Netherlands (Van der Laak et al., 2007).

## 3 Spatial orders in the innovation journey of biofuels

The history of biofuel production and imagination for much of the 20th century is linked to efforts by national governments to secure domestic energy supplies or to support farmers in times of crisis. A number of governments used ethanol for vehicles during World War II (Lewis, 1981). Although petroleum became the dominant basis for automobility after World War II, support for biomass-derived fuels re-emerged in the 1970s around the oil shocks. In this section, we examine the articulation of visions of biofuels since the 1970s, drawing out some key differences in their imagined spatial order and contrasting these with the spatial order of biofuel networks that emerged in the 2000s. We then look at the nature of reflexivity and learning from controversy that biofuels provoked in the early days and more recently.

### 3.1 Niche visions of biofuels compared with global network formation

Niche support for biofuels emerged around policies for agricultural support as well as energy supply. Brazil's sugarcane ethanol programme began in the 1920s as a mechanism for supporting farmers through low sugar prices while in the 1960s, the US state of Nebraska promoted grain alcohol blends (‘gasohol’) as a way of dealing with agricultural surpluses (Bud, 1994; Lewis, 1981). The confluence of energy security and environmental concerns in the 1970s created further impetus for bioenergy policies and research activity to re-emerge more widely in the 1970s–80s. A new journal, *Biomass* (subsequently *Bioresource Technology*) was formed in 1981 and *Energy Policy*, founded in 1973, provided space for bioenergy discussions. *Energy for a Sustainable World* (Goldemberg et al., 1987) outlined a global post-fossil fuel vision with equity, environmental concerns and human needs for energy services at its heart while the influential renewable energy advocate, Amory Lovins, included biomass in his ‘soft’ energy path for the United States. Liquid biofuels were discussed as part of this broader landscape envisioning different applications of bioenergy as sustainable alternative to fossil fuels. Our review of this early history also shows that ‘biofuel’ is not a new word coined to suggest green connotations as some critics have charged (e.g., Action Aid, 2010). The first use of the term in *Energy Policy* occurred in 1989 when it was used to describe biomass-based alternatives to fossil fuel (Gowen, 1989).

In these years after the oil shocks, bioenergy production (including for liquid biofuels) was visualised in the research literature in strongly territorial terms. Just as in wartime, energy security was understood to mean relying on *domestic* resources of biomass to reduce dependence on volatile petroleum *imports*. A key review of bioenergy potential in Western Europe (Hall and House, 1995) focused on domestic biomass; in the one example where biomass imports were discussed, an EU trade deficit in roundwood was mentioned, but this was to acknowledge a potential area of low or non-availability of feedstock for bioenergy. Changing needs and technologies in the industrial roundwood sector were also mentioned to imply a future potential, but there was no suggestion that European energy needs can/would be met by importing biomass. As recently as 2006, the German Green Party (Alliance 90/The Greens, 2006) made a case for biofuels, but their manifesto focused on local biodegradable wastes as feedstock.

By contrast, the spatial order assumed in current biofuel research is distinctly global. Estimates are made of global biomass potential for bioenergy (see Slade et al., 2011), leaving open the option for feedstocks including agricultural residues to be exported/imported. In these current visions, one of the drivers posited for biofuels is ‘rural development’ in the South from growing biomass for export to the world biofuel market. By contrast, in journals such as *Biomass* and *Energy Policy* in the 1980s–1990s, the rural development case for bioenergy was mainly understood as a way of modernising energy services for the poor. This strand on ameliorating energy poverty is now largely distinct from the theme of liquid biofuels, though a few try to re-connect them citing small-scale experiments in producing liquid fuel for cooking or lighting in rural areas (see Nuffield Council on Bioethics, 2011; Sulle and Nelson, 2009). In this context, bioenergy visions are necessarily localised since any technologies would have to rely on resources in the vicinity of rural communities.

Some *non*-territorial future visions do crop up both in the early literature and more recently. Early on, we see glimpses of a vision of international trade in which poorer Southern countries export bio*fuels* as opposed to biomass feedstocks (crops and oils). “There is a long-term practical potential for both Africa and Latin America to become net exporters of biomass fuels such as alcohols and hydrogen” observe Woods and Hall (1994: 4). At present, some middle-income countries do export liquid biofuel, notably, Brazil in ethanol, but also Argentina, India, Indonesia and Malaysia in biodiesel (IEA, 2013). However, African countries remain entrenched as suppliers of raw materials as opposed to higher ‘value-added’ liquid fuel (van Gelder and German, 2011). A second vision for bioethanol is territorialised but in a way that envisions a fairer global trading system for food. Just before biofuels became controversial, there was a proposal from the UK Foreign Policy Centre for large-scale sugar and wheat cultivation for ethanol in Europe as a way of dealing with the inequities of Common Agricultural Policy (CAP) subsidies for European farmers which affect the capacity of small farmers in Africa to compete in the global market (Plesch et al., 2006). They suggested that, for example, Mozambique could develop a lucrative sugar industry if the EU stopped price support for its sugar beet farmers and import duties, and instead supported growing crops for fuel. These two visions have received little attention, and yet, they are relevant for addressing injustices in the global trade system which underlie many of the legitimacy problems of biofuels.

Turning to the biofuel production networks that emerged in the 2000s, we see that some of these were local in nature, inviting little controversy. Conversion of used cooking oil to biodiesel remains non-controversial insofar as it is based on regional schemes (though as we note in Section 4, this too is changing); ‘homebrew’ efforts are promoted by environmental interests (e.g., Journey to Forever) and have been analysed as a form of grassroots ‘citizen technoscience’ (Conz, 2006).^2^ For the most part, biofuel networks have become global (Borras et al., 2010; Franco et al., 2010; Mol, 2007) departing from earlier niche visions and growing around the acquisition of land in the South for sourcing biomass. These investments include both South–South and South–North networks where emerging economic powers, including Brazil and India, have teamed with ‘national’ companies (now themselves transnationalised such as Brazil's development bank) or established multinational companies to pursue biomass supply from poorer Southern countries (Dauvergne and Neville, 2010). NGOs have linked some of these biofuel networks to ‘land grabs’ leading to dispossession of local people and loss of livelihoods (Action Aid, 2010; Friends of the Earth, 2010). The most vulnerable people are bearing the costs of global biofuel development in which a narrative of transforming ‘wastelands’ into green and productive landscapes masks their role as common property resources for the poor (Borras et al., 2010; Dauvergne and Neville, 2010).

**Fig. 1:**
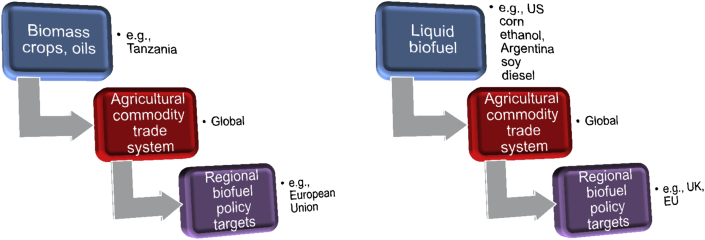
Spatial representation of controversial Biofuel systems.

Policies promoting biofuels are implicated in the formation of this global complex since national and EU targets rely, implicitly or explicitly, on imports of biomass or biofuel rather than domestic supply. Early into the era of UK policy support for biofuels, the House of Commons Environment, Food and Rural Affairs Committee (2003) described government policy as muddled as it failed to clarify whether the aim was to support manufacturing biofuels in the UK using domestically produced feedstocks, or imported feedstocks or, indeed, to simply use imported biofuels, recognising that the spatial orders of a ‘biofuel economy’ can differ. The UK government was responding to the EU's 2003 Biofuels Directive which set a target for biofuels to take up 5.75% of petrol and diesel consumption by 2010, though the *European Biomass Action Plan* (Annex 11) calculated that to reach the target about one-fifth of European tillable land would need to be dedicated to bioenergy crops (Commission of the European Communities, 2005). For this reason, both the *Biomass Action Plan* and the *EU Strategy for Biofuels* (Commission of the European Communities, 2006) noted that Europe will need to source biomass feedstocks for biofuels from beyond its borders. Recent statistics published by the Department for Transport confirm that in order to meet the UK biofuels target of 3.5% of total road transport fuel, imports made up 78% of the reported feedstock (Department for Transport, 2011). Recognising the global spatial order of recent biofuel networks (Fig. 1) can help us begin to understand some of the controversy as we explore below.

### 3.2 Reflexivity in the early stages of the biofuels journey

How far were the controversies of today anticipated by those promoting biofuels soon after the oil shocks?

First, bioenergy promoters in the 1980s–90s did recognise potential threats to food security, particularly for developing countries seeking to reduce petroleum imports by promoting biomass energy on a significant scale (Hall, 1991; Hall et al., 1992; Ramsay, 1985; Rosillo-Calle and Hall, 1992). In the introductory editorial of *Biomass*, the authors write that ‘limitless though the possibilities are [for biomass energy], reality must be kept in sight’ (Coombs and Hall, 1981: 2). They allude to competition with the use of land for food or forestry and the cost of transporting biomass from forests and woodlands to places of production, distribution and use (see also Lewis, 1981). Around the same time, the US Office of Technology Assessment (1980: x), pointed out that “the quantity of biomass that can be obtained on a renewable basis, and the economic, environmental, and other consequences of obtaining it will depend critically on the behaviour of growers and harvesters” and suggested that the use of grains and sugar crops for the production of ethanol might compete with feed and food crop production and lead to rapid inflation in food prices. Goldemberg et al. (1987: 254) made frequent reference to the problem of competition for *good* land between food and fuel and observed that this was already taking place in Brazil.

Some argued that the food-versus-fuel problem was ‘grossly overdrawn’ and that it should be possible to coordinate bioenergy and agricultural production through government intervention or market forces (Ramsay, 1985: 328). Others such as bioenergy pioneer David O Hall (1991) offered one of the most spatially sensitive perspectives of the time while pleading for the right lessons to be learnt from controversies already emerging. Hall observed that food-versus-fuel needs to be considered in the light of rising food surpluses in some parts of the world allied with food *and* fuel shortages in developing countries. He also suggested that use of land for animal feed could be reduced, a factor which has not been considered much in the recent controversy. Where biomass projects have failed, this has been due to a technocratic approach which first prioritises the need for energy rather than a ‘multi-uses’ approach which asks “how land can best be used for sustainable development” (Hall, 1991: 733).

More sceptical views on transport biofuels also emerged in this period including in the Brundtland report (WCED, 1987) which highlighted water pollution from organic waste effluent. Charlesworth and Baker (1978), dismissed biofuel (at that time, methanol or liquefied methane) as a substitute for petroleum-based transport fuels in the UK claiming it would require virtually all of the country's agricultural area. Although describing many renewables as technically promising, Grubb (1990a, 1990b) predicted a minor role for liquid biofuels as indigenous potential in many countries was limited by low conversion efficiency and availability of suitable land. In the US, David Pimentel, highlighted conflicts between agriculture, forestry and energy industries over land and water resources, and increases in land and farm commodity prices. Controversy over the net energy balance of gasohol, an issue that Pimentel continues to highlight, led to US legislation explicitly requiring that the energy content of gasohol products must exceed the petroleum energy inputs in their production (Chambers et al., 1979). Overall, the renaissance of visions of biofuel and other bioenergy technologies in the years after the oil shocks was accompanied by reflexive exploration of criticisms and lessons from practical experience.

**Fig. 2:**
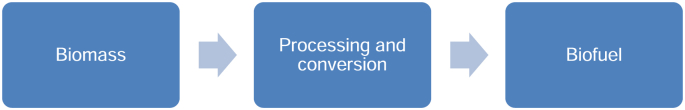
Flat representation of Biofuel system.

Yet, by the early 2000s, the more reflexive discussions seem less evident and biofuel systems were implicitly envisioned in spatially ‘flat’ ways, namely, just a process in which ‘biomass’ of unspecified provenance is converted to fuel (Fig. 2). Renewable energy analysts characterised biofuels as ‘a relatively easy and important carbon-neutral additive to petrol for transport’ (Mitchell and Connor, 2004:, 1942). Biofuels were seen as hindered mainly by policy and market barriers such as high costs, lock-out from existing fuel distribution and storage networks, and a regulatory environment that favoured established suppliers (English et al., 2006; Graham et al., 2000), though this was soon to change with targets set by the 2003 EU Biofuel Directive, the expansion of the biofuel sector and the controversy this then provoked from around 2007. While the key themes in this controversy are widely discussed, we want to explore the nature of lessons drawn and highlight the significance of spatial issues which raise new challenges for globally integrated networks *and* territorial visions of biofuel production from domestic feedstocks.

### 3.3 Reflexivity and learning in the recent biofuels controversy

The most frequently cited concerns about biofuels in the recent debate are threats to food security, practices of ‘land grabbing’, and increases in greenhouse gas (GHG) emissions from indirect land-use change (iLUC) (Nuffield Council on Bioethics, 2011). The role of nitrous oxide emissions associated with fertilisers used in biomass cultivation has also been highlighted with the warning that nitrous oxide is becoming more significant as a greenhouse gas. In addition, the water footprint of biomass cultivation, increased competition for different uses of biomass, wider environmental impacts other than GHG emissions, and local air quality are all mentioned. Some themes only arise at the very margins; for example, the use of antibiotics in ethanol production and the implications for antibiotic resistance is not discernible in major reports assessing the sustainability of biofuels (but see IATP, 2009)) nor is the potential application of agri-biotechnology (but see Levidow and Paul, 2008), though the history of technological controversies suggests that this is not a reason to ignore them.

In the US, the energy balance of ethanol remains contested with some arguing that taking the fossil energy footprint of biomass cultivation into account, the overall energy inputs for ethanol production would be greater than the output (e.g., Gomiero et al., 2010). Given there are different bioenergy applications with varying levels of efficiency, the most *appropriate* use of biomass has been more of an issue in the UK. The point is made that biomass should be reserved for local generation of heat or combined heat and power (CHP) where the energy yield per hectare can be maximised (Clift and Mulugetta, 2007). Higher efficiency and better greenhouse gas savings are possible for bio-heat or CHP, since biomass can be directly burned rather than converted to a liquid, a step that requires further energy inputs. The UK Royal Commission on Environmental Pollution (2004) produced an influential report calling for domestic development of biomass energy, but for CHP rather than transport. The 2007 UK Biomass Strategy (DEFRA, 2007) put liquid biofuels at the bottom of its hierarchy of best use of biomass for energy though it observed that there may still be a case for biofuels in the absence of current alternatives for decarbonising transport.

The criticism of biofuels has more recently been challenged. Pilgrim and Harvey (2010) suggest that NGOs campaigned against biofuels on purely strategic grounds and overstated the negative impacts while the Nuffield Council on Bioethics (2011) observes that the actual role of biofuels in the food price spikes of 2007/08 is contested. Ethanol producers argue that indirect land-use change (iLUC) is too complex and that current ways of modelling it too unreliable for policymaking purposes (ePURE, 2011). In this context, it is important to clarify what exactly is at stake in the assessment of biofuels, lessons of spatial difference which are obscured in generic narratives of food versus fuel or local versus global, or in the use of economic models to determine the relevance of biofuels to food security and land-use change, or indeed, on the strategies adopted by specific groups.

First, the problem of spatial unevenness of sustainable technologies is clearest around land acquisition in the global South, often to the detriment of local communities, for biofuel use in the North. The impact of ‘land grabs’ has been widely explored in development studies and activism and, to a lesser extent, in *Energy Policy* where the power of multinational companies to impact local livelihoods by consolidating small holdings has been highlighted (e.g., Tomei and Upham, 2009; van Eijck and Romijn, 2008). But land-use challenges are not only a North/South problem – they are now made to matter within national borders around territorialised policy visions for biofuels. As human/economic geographers argue, the idea that sustainability projects can exist at a discrete and homogeneous national level is problematic (Coenen et al., 2012; Swyngedouw, 2007). For example, Brazil's ethanol programme is criticised for its unequal power relations and uneven environmental impacts. Lehtonen (2011) describes the influence of industrial and landowning elites in the Northeast, the disproportionate environmental burdens from sugarcane processing and ethanol production on poor rural populations in Northeastern Brazil by comparison with improvements in air quality for Southeastern urban middle classes where the ethanol is used, and harsh working conditions in sugarcane plantations (see also Franco et al., 2010). As Brazil has become a leading exporter of ethanol, pressures to adopt large-scale mechanised farming practices also means that small farmers become excluded from potential benefits (Hall et al., 2009).

Another example of the limits of national/regional visions is the government of India's target of 20 percent biomass in domestic diesel supply by 2010–11. Regional governments in India promoted biofuel production in partnership with MNCs, many around *Jatropha curcas*, an oilseed lauded as growing on ‘marginal’ land or ‘wastelands’. The policy became controversial as conflicts emerged between official designations of ‘marginal’ land and the livelihood, food and fuel needs of the rural poor living there, and encroachment of plantations into agricultural or irrigated land where fuel yields are higher (Ariza-Montobbio et al., 2010).

Second, and in contrast to the post-1970s promotion of bioenergy, the uneven sustainability impacts of biofuel production are now understood in global terms, adding to the de-legitimisation of national visions. We see this in the two most prominent concerns about biofuels' impact on food security and greenhouse gas emissions. But since the global is often presented in impersonal and overarching terms, it is important to understand how global networks are linked to local nodes where their impacts are experienced and sometimes resisted.

Taking ‘food-versus-fuel’ first, the problem is frequently represented in terms of economic modelling of global food prices, specifically, factors relating to price spikes in 2007/08 and predictions of future rises. While an OECD/FAO (2007) policy briefing warned of sustained high food prices and some predicted doubling of food prices in some countries by 2050 from pressure created by biofuels (Johansson and Azar, 2007), the Nuffield Council on Bioethics (2011) concluded that blaming biofuels for the price rises is one-sided, and that the role of biofuels was smaller than other factors such as high energy prices and the weak dollar. Yet, given the complexity of a highly networked global economy, this is not surprising. The UK Renewable Fuels Agency (2008) acknowledged that the effect of biofuels on food prices is complex and difficult to model, but observed that the poorest people (amongst the category of those relying on the market for food) are likely to be the most adversely affected. Likewise, the OECD/FAO (2007) emphasised the impact on developing countries that are net food importers.

These points suggest that the stakes over ‘food-versus-fuel’ cannot be settled by global models that erase local differences. Extending a ‘local node, global network’ perspective (Coenen et al., 2012), the pertinent questions are *which* land, *whose* food and *whose* fuel sources are at stake. First, if biofuels are putting *some* pressure on food prices, the fact that it is part of a structural regime of interconnected factors makes it no less of a problem for people who are most vulnerable to even small price changes. In this context, national biofuel visions (as, for example, the US pursuing energy security by converting parts of its corn belt for ethanol production) may be challenged if they are perceived to have knock-on effects elsewhere (as when the US corn ethanol programme was seen to impinge on people in Mexico around the so-called ‘tortilla riots’). Second, subsistence farmers may not see a conflict between food and fuel, but if they were displaced by biofuel plantations from the land off which they live, the conflict between the fuel needs of distant peoples and their own needs for food *and* fuel becomes stark. Food/fuel conflicts may also emerge if small farmers were induced by large foreign investors to convert prime cropland for higher-yield biofuel cultivation or to sell their land for short-term gain at the expense of established sources of livelihood and food/fuel supply (van Eijck and Romijn, 2008). Such connections between different spaces and scales of biofuel and food production specify the potential conflict in more compelling ways than is captured by a global model.

The broader environmental credentials of biofuels have also become controversial, but here again, the spatial locus of sustainability assessments is not fully acknowledged. Two widely cited studies highlighted increases in greenhouse gas emissions from direct and indirect land-use changes. Fargione et al. (2008) noted that biofuels incur a carbon debt if rainforests, peatlands or grasslands are converted to grow food crops, thereby releasing large carbon stocks into the atmosphere. Searchinger et al. (2008) argued that increases in corn-based ethanol in the US would lead to indirect land-use change (iLUC) worldwide with farmers diverting existing cropland to biofuels which in turn triggers higher crop prices and further conversion of forests and grasslands globally for food production to replace the diverted land. The release of carbon stocks in such cases, though strictly related to land use for food rather than fuel, should be counted, it was argued, as they were triggered by cultivation for biofuel. Such studies are again based on modelling, and their assumptions have been contested (e.g., Kline and Dale, 2008). As in the modelling of biofuel impacts on food prices, the focus has been on the *net* GHG implications of biofuels. From a spatial perspective, however, land-use studies are more significant for helping to make visible the ways in which the biofuels project (like other technological transitions) creates new connections between diverse locales with uneven impacts on sustainability.

**Fig. 3:**
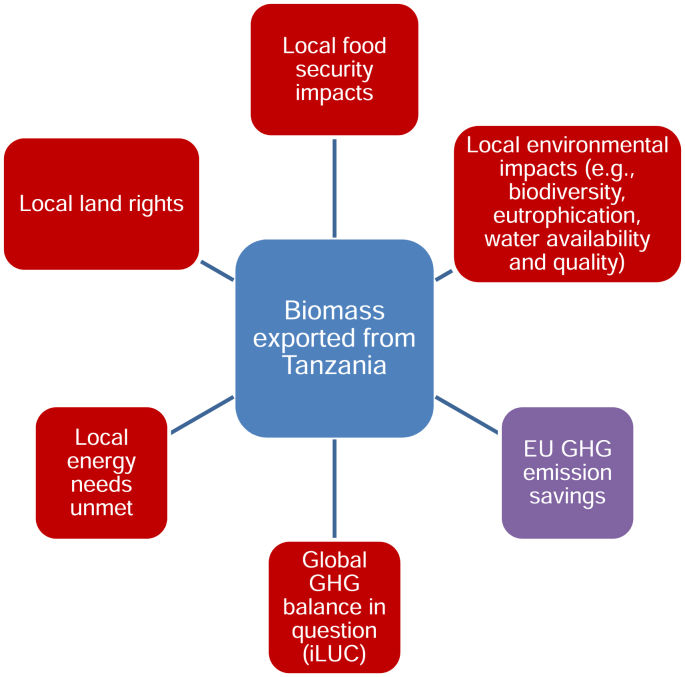
Spaces of Biofuel sustainability impacts (biomass export).

**Fig. 4:**
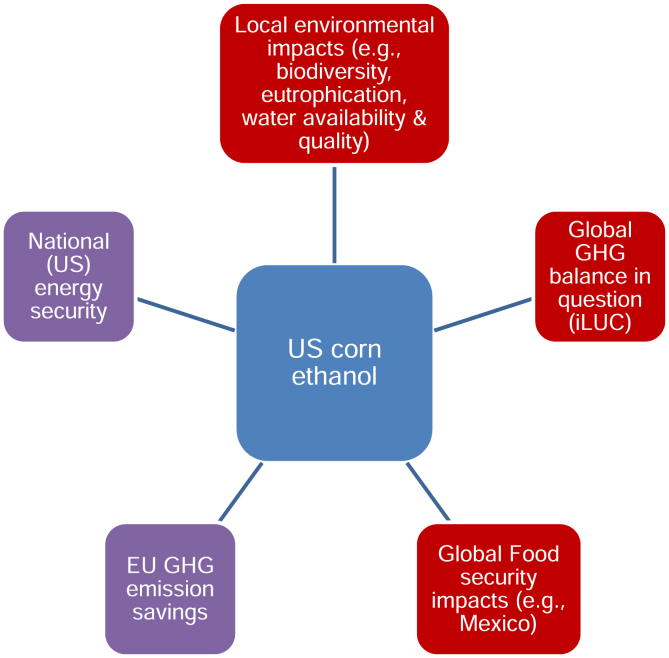
Spaces of Biofuel sustainability impacts (biofuel export).

In sum, while biofuels have been interrogated intensively in recent years, the precise lesson drawn from this controversy seems to be pitched at a generic level of ‘food-versus-fuel’ or net GHG impacts from land use changes to be assessed mainly by modelling. The more fundamental point that biofuel impacts on food security or the environment are likely to vary across locales – and that global models do not readily capture this - is not really discussed. Figs. 3 and 4 highlight the need to understand the spatial ordering of sustainability impacts. Negative environmental impacts of biomass cultivation in Tanzania (for example) will be experienced locally, though those countries importing the biomass or biofuel produced from it benefit in terms of their greenhouse gas balances. Parts of the US may similarly experience environmental risks associated with their corn ethanol programme but so might people elsewhere affected by changes in food markets.

### 3.4 Reflexivity beyond the modelling of biofuel impacts

As biofuel visions and networks continue to be interrogated, opportunities for considering these more fundamental questions about sustainability beyond those raised by quantitative modelling are gradually opening up. Some proponents of biofuels argue that biofuels are unfairly demonised while the use of land for food and other non-food goods escapes scrutiny (Wenner, 2012). It is therefore important to consider the wider industrial–agricultural system of which biofuels are a part. What are the lessons from the biofuels controversy if we were to take this whole system into account?

First, the notion that biofuels are the *only* agri-technology to be criticised is difficult to substantiate as social movements have campaigned against global production systems for a number of agricultural commodities such as cotton, cocoa, sugar and coffee. ‘Fairtrade’ alternatives emerged from a history of campaigning against unequal trading arrangements, represented most recently by protests against the World Trade Organisation (WTO) model of globalisation seen in 1999 in Seattle and afterwards. Well before the biofuels question, farming practices and policies in the EU and elsewhere were subjected to sustainability challenges in debates around the reform of the Common Agricultural Policy (CAP). Biofuels are therefore only the most recent case in a longer trajectory of political, environmental and ethical questions raised about agricultural systems. There are some hints of this in the earlier promotion of bioenergy; for example, David O Hall (1991) noted that fertiliser inputs and monocultures need to be reduced or avoided in order to ensure biodiversity. Similar concerns about monoculture plantations are echoed in recent critiques by NGOs (Action Aid, 2010; Smolker et al., 2008).

While the challenges outlined above continue to be live issues, the relationship between biofuels and food is also being articulated in new ways, highlighting further spatial aspects that are ignored in generic ‘food-versus-fuel’ arguments. The first of these illuminates the place of biofuels within the political economy of industrial agriculture, implying that some of the problems attributed to biofuels are partly a problem of this wider system. Writing on the 2007 ‘tortilla riots’ which came to symbolise the biofuel controversy, Walden Bello asks: ‘how on earth did Mexicans, who live in the land where corn was domesticated, become dependent on US imports in the first place?’ (Bello, 2008, p.1). Bello details the impact of World Bank/IMF structural adjustment policies of the 1980s and the 1994 North American Free Trade Agreement (NAFTA) which mandated the removal of import tariffs and pitted Mexicans farmers against cheap US corn imports. If subsequent diversion of US corn to ethanol caused higher corn prices and affected poor Mexicans, these governance structures were at least partly to blame.

The ‘food-versus-fuel’ framework has also been questioned by those highlighting the place of the livestock industry and meat consumption within the food system. Some suggest that future conflict may not be between ‘cars and the poor’ but between ‘cars and carnivores’ (de Fraiture et al., 2008: 79). Wassenaar and Kay (2008: 201) argue that ‘researchers [should] stop presenting bioenergy as an aggressive intruder on an agrarian utopia’, that ‘not all current forms of land use are critical to society’ and that the animal feed industry is a particular problem. The use of land and, in particular, grain for animal feed has generated what was described in 1987 as a post-Malthusian ‘food versus feed’ problem with the rise of a global market for food and feed and the growth of middle-income, meat-consuming classes (Yotopoulos, 1985). Some now advocate biofuels on the basis of reductions in the use of land for animal feed, a point prefigured in David Hall's earlier assessment of bioenergy (1991: 733). Known for promoting grassroots experiments in sustainable living, the UK Centre for Alternative Technology's (CAT) vision of a Zero-Carbon Britain by 2030 assumes a dramatic reduction in the use of land for animal grazing (and, in turn, meat consumption) suggesting that this land could be released for producing biomass-derived energy including biofuels (Centre for Alternative Technology, 2010). In this case, the space of food production is no longer sacrosanct or exempt from critical scrutiny of its own sustainability credentials, though biofuel production and use are once again envisaged in national-territorial terms.

The return to space in the reframing of biofuels is more explicit on the website of *Journey to Forever* (http://journeytoforever.org), a self-described mobile, environmental NGO which questions the food-versus-fuel dynamic by referring to wider systemic causes of hunger. It seeks to resurrect biofuels by distinguishing the use of locally available resources for local use from ‘agrofuels’. ‘Objections to biofuels-as-agrofuels are really just objections to industrialised agriculture itself, along with “free trade” (free of regulations) and all the other trappings of the global food system that help to make it so destructive’, they argue. Prominent NGO critiques of biofuels (Action Aid, 2010; Friends of the Earth Europe, 2010; Smolker et al., 2008) are on similar lines, employing the language of ‘agrofuels’ to highlight the problems of biofuels as an output of globalised industrial agriculture. Christian Aid (2009) summarise the issue succinctly: “*The problem is not with the crop or the fuel – it is with the policy framework around biofuel production and use*” (p.4). In asking why biofuels have been targeted when other agricultural technologies and uses of land apparently have not (Wenner, 2012), we find that the biofuels controversy draws attention to precisely this wider agricultural system.

## 4 Discussion and conclusions

In this paper, we have shown why and how spatial connections and spatial unevenness are important for understanding the ‘riches to rags’ journey of biofuels. Contemporary concerns over food security were anticipated by those promoting bioenergy in the 1980s; however, it was expected that these could be managed at the local or national level from where biomass resources would be sourced. Where the territorial vision was breached, it was to imagine Southern countries benefiting by exporting higher-value biofuel to the North, an option that has not materialised with the exception of Brazil. By contrast, the current controversy can be traced in part to the growth of a globally integrated biofuel network in which the poorer parts of the South have featured mainly as feedstock suppliers (Mol, 2007; van der Horst and Vermeylen, 2011).

Our historical analysis leads us to an important question. If bioenergy was originally meant to be a territorially based technology, could a domestic system of biofuel production with countries growing biomass for fuel within their own territories help re-legitimise biofuels? Our analysis suggests the need for caution. Space now matters in other important respects that were less recognised in the earlier era of bioenergy; these include conflicts *within* national territories as well as those sparked by national biofuel systems having significant impacts beyond their territorial boundaries. More work is needed on these spatial linkages which are not exhausted by the North/South connections highlighted in recent literature (Mol, 2007; van der Horst and Vermeylen, 2011; Journal of Peasant Studies, 2010 special issue). For the moment, we outline four key issues.

First, land conflicts and uneven environmental impacts of biofuel production are more evident within countries such as Brazil and India (where they are built in part through multinational linkages) as well as across global networks. Second, so long as the agri-food system is global in nature, territorial production of biomass for fuel can still have impacts beyond national borders as seen in the iLUC controversy over the global impact of ‘domestic’ US investment in corn ethanol discussed in section 3.3. Third, the economic and environmental challenges of *land* transportation of biomass for energy production at some distance from the biomass source is starting to be highlighted by some in the bioenergy community (Sanderson, 2011). The significance of space for biofuel sustainability therefore extends to conflicts within territories; it has been suggested, for example, that carbon emissions from trucks used to transport pellets over large distances within the US are more significant than shipping emissions associated with Atlantic trade (Chazan, 2013). Fourth, *North/North* conflicts around biofuels are also emerging; for example, the European Union imposed anti-dumping duties on imports of US biodiesel citing the unfairness of government subsidies and the European Commission has recently proposed similar restrictions for US ethanol (ICTSD, 2013).

What then are the lessons for those who are promoting alternative biofuel visions, either from biodegradable wastes or from non-edible feedstocks (so-called second generation biofuels) (Mohr and Raman, 2013)? The case for second-generation (2G) biofuels has been largely made on the basis that it avoids a food-versus-fuel conflict. However, the first-generation journey shows that the problems of biofuels are more complicated than implied by a generic conflict with food. Rather, they arise from a globalised system with a spatially uneven distribution of sustainability risks and benefits. These challenges are likely to remain insofar as biofuel feedstocks and systems of production are part of the global agrarian economy. Likewise, in response to concerns that it is the inefficiency of biomass processing for liquid fuel (Clift and Mulugetta, 2007) that is the real problem, the same controversies that have affected biofuels are likely to arise where biomass is imported for more efficient bioenergy applications. We are now seeing this with UK protests over proposed power stations that would use imported palm oil, other vegetable oils or wood pellets (BiofuelWatch, 2011). Nor can we assume that the use of biodegradable waste such as used cooking oil (UCO) automatically circumvents controversy. UK biofuel statistics show that this too is a commodity that is being traded across borders. Since UCO can be double-counted towards the biofuel targets set by the amended EU Renewable Energy Directive and since it continues to have UK duty subsidies, prices have risen, and there are concerns about lack of traceability and monitoring procedures and incentives for un-used oils being passed off as waste (IEA Bioenergy, 2013). The spatial order that second-generation biofuel or fuel-from-waste takes in practice is therefore crucial.

New ways of thinking about the food–fuel relationship are emerging that challenge the assumption that there is an intrinsic conflict between the two. The more contested aspects of food production (especially for animal feed), land use for other non-food goods, or the need for fuel to produce food (Karp and Richter, 2011) should help future debate be placed in the wider context of land-use policies as a whole. But spatial arrangements and the rules of global agricultural trade will remain important in this context as a generic food-and-fuel synergy may spotlight the problems of global industrial agriculture even *more*. In this respect, some alternative visions which appear to challenge the rules of this global system could be significant for addressing legitimacy issues.

First is the vision for large-scale sugar and wheat cultivation in Europe for ethanol (Plesch et al., 2006) which has been proposed as a way of changing the inequities of Common Agricultural Policy (CAP) food subsidies (e.g., for crops such as sugar beet) that affect the capacity of small farmers in the South (e.g., sugar producers such as Mozambique) to compete in the global market. Here, producing fuel in place of food is seen as a corrective to trade injustices. It contrast to the US experiment with corn ethanol, this territorial vision for biofuels would require changes to the rules and balance of power within world trade. Second, some US biofuel advocates are trying to stimulate debate on fundamental issues of ownership structures in agriculture and world trade negotiations for a ‘better, decentralised biofuel model’ (Morris, 2005). Third, some working in the global South are exploring small-scale biofuel models for addressing local energy poverty (Martin et al., 2009; van der Horst and Vermeylen, 2011), and conditions under which smallholder projects in Tanzania producing biomass for export could be viable (Sulle and Nelson, 2009). Given the uncertainties over the capacity of sustainability certification schemes to manage the current problems of biofuels (Tomei and Upham, 2009), these alternative visions and experiments might be promising.

Finally, what are the implications of our analysis for sustainability assessment which has been the main way through which the biofuel community has responded to the controversy? Sustainability assessment methods play an important role in identifying key challenges of new technologies across the ‘whole system’, giving an indication of the relative environmental significance of different aspects of production, and giving recognition to a range of different criteria beyond the strictly ‘environmental’ alone. But as Palmer (2012) argues, when sustainability metrics have been used to enact biofuel policies, the underlying political questions have been prematurely foreclosed. We have suggested that these questions relate to the legitimacy of globalised industrial agricultural systems as such. Sustainability assessment needs to be informed by and put in context of these wider issues in order to do justice to the challenges.

In conclusion, our analysis of biofuels demonstrates the value of looking at how visions, networks and learning around new technologies are articulated and reshaped over time as suggested by sustainable innovation journey research (Geels et al., 2008). Following work in human geography (Coenen et al., 2012; Swyngedouw, 2007), we have also shown the importance of bringing space to bear on the understanding of sustainable innovation journeys. The historical approach adopted in this paper helps bring out the original distinctiveness of bioenergy as a territorialised energy technology, a vision that could be revisited in current debates about biofuel futures. However, the addition of a spatial perspective means even local/national systems may be linked to global networks and have impacts beyond their territorial boundaries, generating North–North conflicts (as seen in EU anti-dumping duties imposed on US biodiesel) and South–South conflicts (as seen in as well as the North–South conflicts that underlie the recent controversy. How these linkages and the ethical/political questions they raise are managed will be crucial for the future of biofuels. Here, a promising novel line of investigation on sociotechnical imaginaries (Jasanoff and Kim, 2009) could be developed to explore how state and state-like entities channel investment in bioenergy projects which are spatially ordered in particular ways as opposed to others, define how these investments constitute the public good, and justify the inclusion/exclusion of specific publics in their governance (e.g., Levidow, 2012).

## Footnotes

1 We acknowledge the contribution of Kate Millar, our collaborator on the BBSRC LACE project, who helped frame this question.

2 Journey to Forever is described as a mobile NGO working on local environment and development projects. http://www.journeytoforever.org/.
